# SelSA-1, a novel HDAC inhibitor demonstrates enhanced chemotherapeutic potential by redox modulation

**DOI:** 10.1038/s41598-023-36555-w

**Published:** 2023-06-08

**Authors:** Ayushi Garg, Dhimant Desai, Aman Bhalla, Shalu Thakur, Pulkit Rastogi, Naveen Kaushal

**Affiliations:** 1 Department of Biophysics, Panjab University, Chandigarh, 160014 India; 2 Departments of Pharmacology, Pennsylvania State University College of Medicine, Hershey, USA; 3 Department of Chemistry and Centre of Advanced Studies in Chemistry, Panjab University, Chandigarh, 160014 India; 4 Department of Hematology, Post Graduate Institute of Medical Education and Research (PGIMER), Chandigarh, 160012 India

**Keywords:** Biophysics, Cancer, Cell biology, Molecular medicine, Oncology

## Abstract

Colorectal cancer (CRC) is a multistep disorder resulting from genetic and epigenetic genome changes. It is the third most common malignancy in developed nations accounting for roughly 600,000 deaths annually. Persistent gut inflammation, as observed in inflammatory bowel disease (IBD), is a key risk factor for CRC development. From an epigenetic viewpoint, the pharmacological inhibition of HDACs using HDAC inhibitors such as SAHA has emerged as a suitable anticancer strategy in the recent past. However, the clinical success of these strategies is limited and has risk factors associated with their uses. Thus, considering the critical involvement of epigenetic regulation of key molecular mechanisms in carcinogenesis as well as HDAC inhibitory and anti-tumorigenic properties of Selenium (Se), we aimed to explore the potentially safer and enhanced chemotherapeutic potential of a Se derivative of SAHA namely SelSA-1, in an experimental model of colitis-associated experimental cancer (CAC) model and mechanism involved therein. The in vitro study indicated improved efficiency, specificity, and better safety margin in terms of lower IC_50_ value of SelSA-1 than SAHA in both NIH3T3 (9.44 and 10.87 µM) and HCT 115 (5.70 and 7.49 µM) cell lines as well on primary colonocytes (5.61 and 6.30 µM) respectively. In an in vivo experimental model, SelSA-1 efficiently demonstrated amelioration of the multiple plaque lesions (MPLs), tumor burden/incidence, and modulation of various histological and morphological parameters. Further, redox-mediated alterations in apoptotic mediators suggested induction of cancer cell apoptosis by SelSA-1. These findings indicate the enhanced chemotherapeutic and pro-resolution effects of SelSA-1 in part mediated through redox modulation of multiple epigenetic and apoptotic pathways.

## Introduction

Selenium (Se), an essential micronutrient and established antioxidant has been long known for its chemo-preventive properties. Numerous investigations have established an inverse correlation between dose, forms, and extent of Se administration with a wide range of incidence and severity of countless intestinal diseases such as colorectal cancer (CRC) and inflammatory bowel disease (IBD)^1^. For example, low Se intake exacerbates colitis, promotes inflammation-associated carcinogenesis, and has been considered a major risk factor related to CRC^2^. Contrary to this adequate or supra-nutritional concentrations of Se have been associated with anticarcinogenic, therapeutic, and resolution properties. Studies revealed that such effects of Se are arbitrated through modulation of various selenoproteins (the biological actors of Se) such as glutathione peroxidases (GPx), and thioredoxin reductases (TR) that exhibit oxidoreductase activities by attenuating oxidative stress^3,4^. Such epidemiological and clinical evidence along with the redox modulatory anti-tumorigenic efficacy of Se has led to significant interest and exploration of numerous Se-containing compounds or seleno derivatives of conventionally available drugs as cancer therapeutics^5,6^.

Diverse epigenetically regulated mechanisms such as DNA methylation, microRNA, and histone modifications are involved in carcinogenesis^3^. Histone acetylation involving Histone acetyltransferases (HATs) and Histone deacetylases (HDACs) is one such histone modification that can alter the gene transcription and activities of various histone and non-histone proteins during the process of angiogenesis, apoptosis, and carcinogenesis^7^. From a gastrointestinal viewpoint, HDACs in particular as transcriptional regulators can influence colon cell maturation, differentiation, and transformation^8^.

Considering the critical role of HDACs in tumorigenesis, a series of epigenetic therapeutics inhibiting these HDACs are being developed, including natural (Trichostatin A and the depsipeptide FK228) and synthetic (hydroxamic acids, carboxylic acids, benzamides, epoxides, and cyclic peptides) HDAC inhibitors (HDACi). These HDACi inhibit cancer cell growth by stimulating cellular differentiation and apoptosis. Despite this understanding, to date, there are only a few clinically approved HDACi drugs such as Vorinostat, Romidepsin, Bexarotene, Belinostat, and Mogamulizumab for different treatment modalities^9,10^. Vorinostat [also known as Suberoylanilide Hydroxamic acid (SAHA)], is one of the above mentioned broad-spectrum HDACi, which is FDA-approved for the treatment of cutaneous T-cell lymphoma^9^. However, SAHA was, unfortunately, less effective against solid tumors and induces severe side effects^11,12^. Thus, considering the chemotherapeutic limitations of available HDACi such as SAHA and associated toxicities when used at higher concentrations warrants the need for a more efficient, consistent chemotherapeutic drug with innocuous profiles that would be useful as part of an epigenomic-based treatment strategy^13^.

Reports, including studies from our laboratory, have previously indicated that Se through regulation of various epigenetic events viz histone modifications has promising anti-inflammatory and anti-tumorigenic effects^14,15^. Se, through regulated acetylation reduction of H4 at K16 and K12 residues at the promoter region of genes like COX-2 and TNF-α, can exhibit critical anti-carcinogenic potential^16^.

Therefore, based on the emerging evidence from epidemiological studies and clinical trials showing the beneficial anti-inflammatory and chemo-preventive effects of Se^17,18^ and Se-based drugs^14,19^, currently, we report the attenuation of the SAHA-associated toxicity and improvement in its chemotherapeutic profile by the Seleno-derivative of SAHA namely SelSA-1 in Azoxymethane/Dextran Sulfate Sodium (AOM/DSS)-induced experimental colitis-associated colon cancer (CAC) model. Further, the potential redox modulatory chemotherapeutic mechanism culminating in enhanced apoptosis was explored**.**

## Results

### SelSA-1 shows better efficacy and safety profiles than SAHA

**Figure 1:**
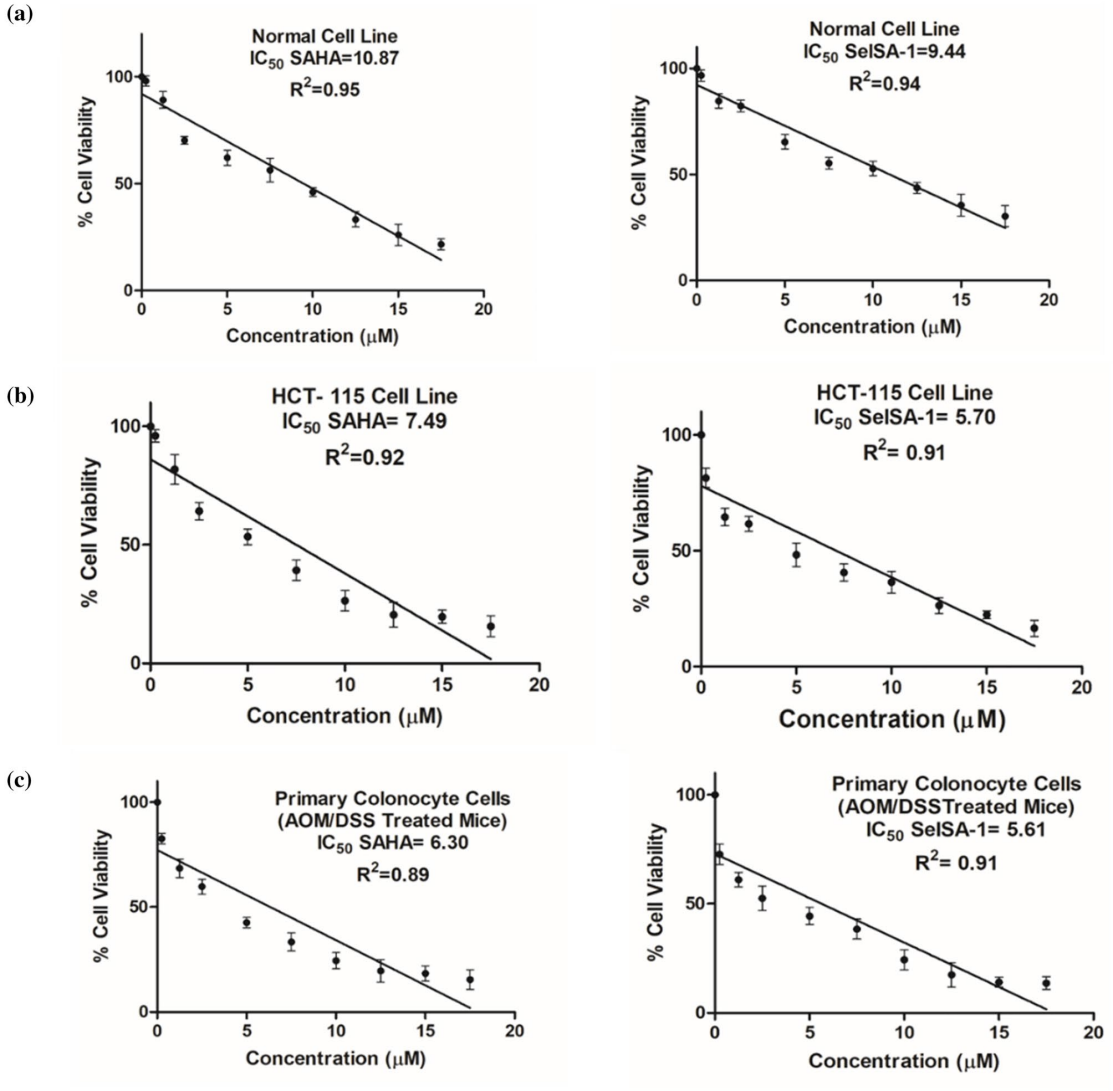
Linear regression plot for the determination of IC_50_ values of SAHA and SelSA-1 against (**a**) NIH3T3 (Normal cell line) (**b**) HCT-115 (Colon cancer cell line), and (**c**) Primary Colonocytes cells isolated from AOM/DSS treated mice. A μM range of concentration of SAHA and SelSA-1 drug were examined for 24 h to determine the respective IC_50_ values. Results represent the mean ± SD value of three independent experiments, each performed in triplicates.

Cell viability and IC_50_ values for normal, cancer cell lines and, primary colonocytes cells were measured following the treatment with different concentrations 0.25 µM, 1.25 µM, 2.5 µM, 5 µM, 7.5 µM, 10 µM, 12.5 µM, 15 µM, 17.5 µM of respective drugs i.e., SAHA and SelSA-1. As depicted in Fig. 1a–c, Supplementary Fig. S1, and Table 1, SelSA-1 demonstrated better efficacy in terms of lower IC_50_ values and better safety margins in terms of cell viability as compared to that of SAHA in both the cancerous primary colonocytes cells isolate from AOM/DSS treated mice and HCT-115 cell lines. The IC_50_ values of SAHA and SelSA-1 in the case of HCT-115 cell line were found to be 7.89 and 5.70 µM respectively. Similarly, in the normal cell line (NIH3T3), although the IC_50_ values for SAHA and SelSA-1 were 10.87 and 9.44 µM respectively, therefore there was no significant difference (p ≤ 0.05) observed in the IC_50_ values as shown in Fig. 1a, and Table 1.

**Table 1:** Shows the IC_50_ values of SAHA and SelSA-1 after 24 h determined through the linear regression plot of percentage cell viability against the various concentrations of the drug.

Types of cells	IC_50_ (µM) at 24 h	
	SAHA	SelSA-1
Normal cell line NIH3T3	10.87 ± 0.965	9.44 ± 0.715
Colon cancer cell HCT-115	7.49 ± 0.515	5.70 ± 0.48
Primary colonocytes from AOM/DSS treated mice	6.30 ± 0.385	5.61 ± 0.415

Results represent the mean ± SD value of three independent experiments, each performed in triplicates.

### SelSA-1 promotes the resolution of colonic inflammation in CAC model

**Figure 2:**
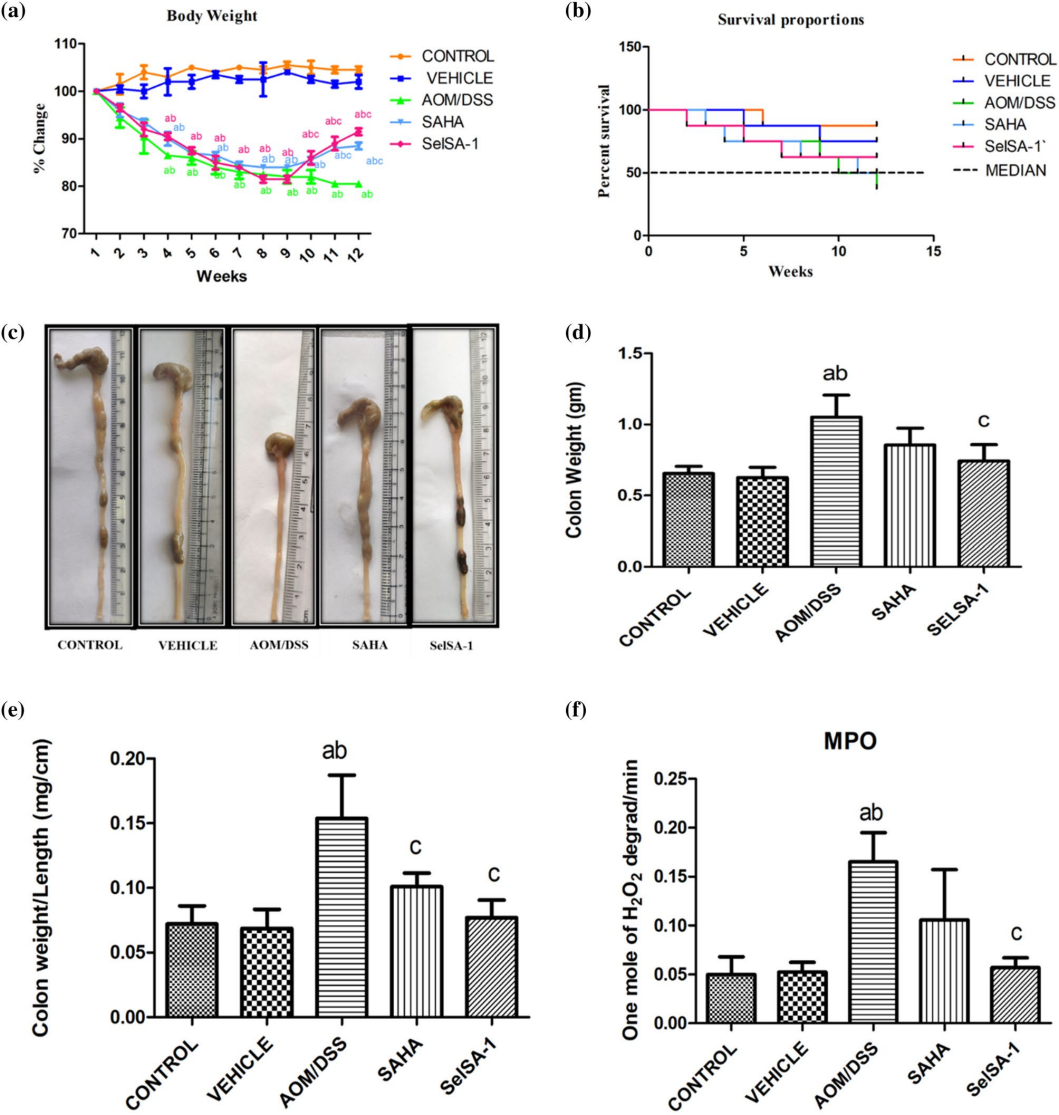
Demonstrate the variations in the classical indices of colitis-associated insult (**a**) Body weights (% change), (**b**) Survival Curve (% change), (**c**) Pictorial representation of Colon Length shortening, (**d**) Colon weights, (**e**) Colon Weight: Colon Length Ratio, (**f**) MPO activity in mice after AOM/DSS treatment respectively in five different groups. Data are expressed as mean ± SD of 7–8 independent observations and analyzed using one-way ANOVA (Turkey multiple comparison methods) where (**a**) represents p < 0.05 when compared between Control *vs* Vehicle treated, AOM/DSS, AOM/DSS + SAHA, AOM/DSS + SelSA-1; (**b**) represents p < 0.05 when compared between Vehicle *vs* AOM/DSS, AOM/DSS + SAHA, AOM/DSS + SelSA-1; (**c**) represents p < 0.05 when compared between AOM/DSS *vs* AOM/DSS + SAHA, AOM/DSS + SelSA-1; (**d**) represents p < 0.05 when compared between AOM/DSS + SAHA *vs* AOM/DSS + SelSA-1.

Changes in body weights, survival rates, and colonic lengths are classical indices of colitis-associated insult. Figure 2a shows the body weights of mice throughout the experimental period. A continuous time-dependent loss in the body weight of mice after AOM/DSS (Group 3) administration was observed. Although, the body weights in AOM/DSS + SAHA and AOM/DSS + SelSA-1 (Group 4 & 5) animals remain lower at the end of the experimental protocol than that of the Control and Vehicle (Group 1 & 2) group, but it was significantly recovered (p ≤ 0.05) than those in AOM/DSS administrative groups. A clear recovery of body weights of animals in the group i.e., AOM/DSS + SAHA, and AOM/DSS + SelSA-1 groups was observed.

Along similar lines, the percent survival of different animals depends upon their body weight after AOM/DSS administration. The Kaplan–Meier curve Fig. 2b, showing the percentage survival of animals indicated that animals with AOM/DSS administration had poor survival rates (38%) compared to other treated groups such as SAHA (50%) and SelSA-1 (66%) respectively.

Further, colitis-associated shortening of colon lengths was observed in AOM/DSS group, when compared to the normal Control and Vehicle treated group Fig. 2c–e. Contrary to this, the treatment with SAHA and SelSA-1 shows a sign of normalization with a substantial recovery (p ≤ 0.05) in the colon length and colon weight, when compared with alone AOM/DSS group, whereas animals with SelSA-1 indicated a better resolution than SAHA as shown in Fig. 2c–e. Likewise, MPO activity as a biochemical marker of inflammation indicated activated neutrophils infiltration in the AOM/DSS-induced colonic inflammation in comparison to the Control and Vehicle-treated group as shown in Fig. 2f. SAHA treatment reduced the inflammation in the colon, but it was not statistically significant when compared to the Cntrol and Vehicle-treated group. On the other hand, SelSA-1 treatment led to a statistically significant reduction (p ≤ 0.05) in the MPO activity indicating effective amelioration of inflammation by SelSA-1 as indicated in Fig. 2f.

**Figure 3:**
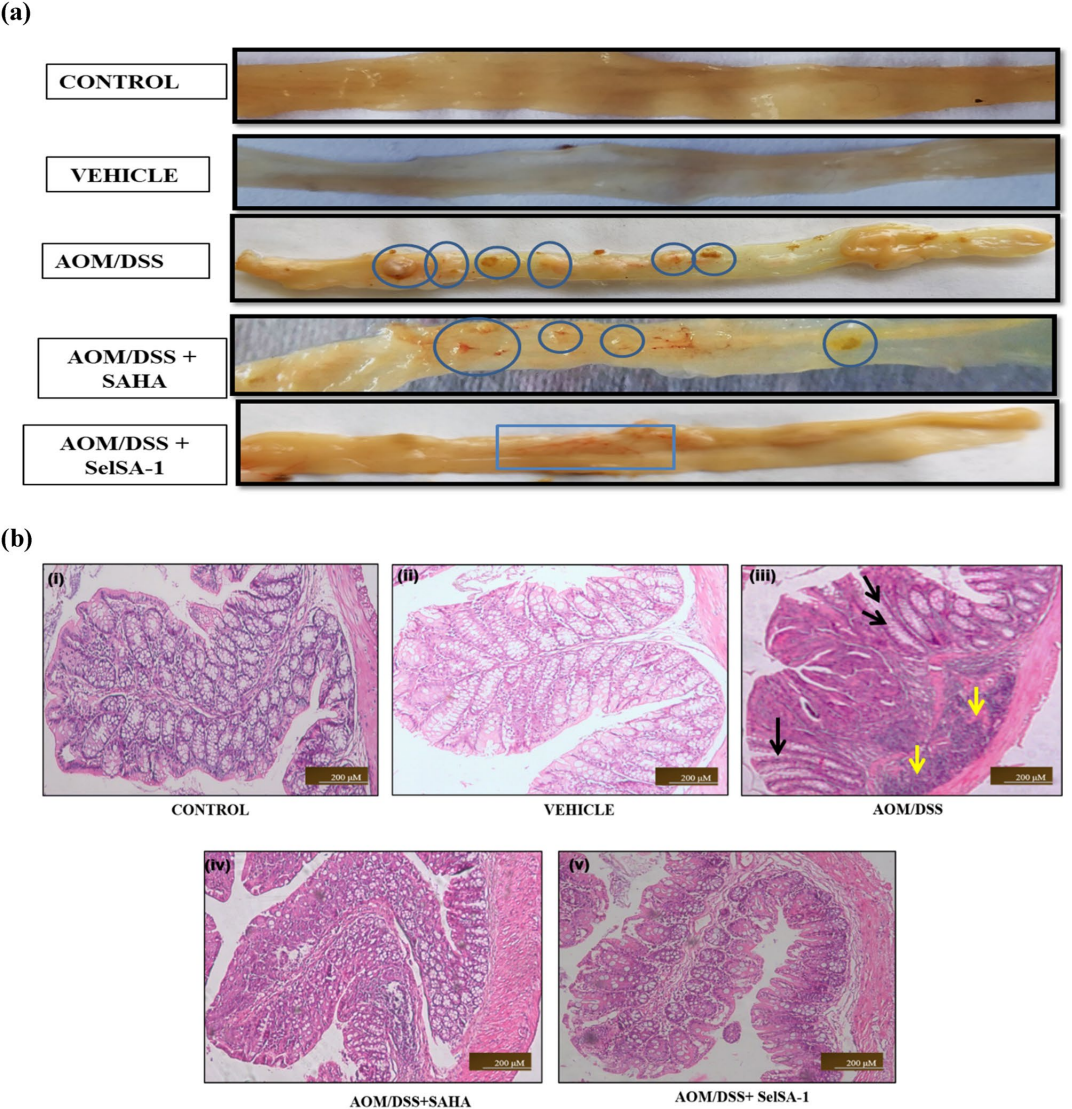
Shows gross morphological and histopathological changes in the colon tissue of different experimental groups. (**a**) AOM/DSS group shows more tumor/lesions (as shown by blue circles) as compared with Control and Vehicle-treated showing normal morphology while SelSA-1 intake has significantly reduced these lesions. (**b**) Photomicrographs illustrating the histopathological alterations at 10 × after 12 weeks in the colonic segment of different groups, respectively. The carcinogenic group compared to the Control and Vehicle-treated group shows severe epithelial disruption hence more neutrophils influx (as shown by yellow arrows) as well as more crypt distortion (as shown by black arrow). However, administrating SAHA and SelSA-1 ameliorated these carcinogenic variations.

Morphological analysis in Fig. 3a depicts maximum morphological insult with signs of inflammation and the presence of neoplastic lesions in the AOM/DSS group as compared to the Cntrol and Vehicle-treated group, where no such symptoms were observed. Also, other morphological indicators such as MPLs were found to be increased in AOM/DSS group (neoplastic lesions marked as blue circles), when compared with other treated groups Table 2 and Fig. 3a. Whereas AOM/DSS group indicated 100% tumor incidence, SAHA treatment reduced the tumor incidence to 62%, whereas SelSA-1 showed maximum chemotherapeutic potential with a reduction in tumor incidence to 37.5% (Table 2) as well as tumor burden and multiplicity. These findings established the enhanced in vivo chemotherapeutic efficacy of SelSA-1 than classical 2nd generation HDACi SAHA.

**Table 2:** Evaluation of multiple plaque lesions (MPLs) in the form of tumor burden, multiplicity, and incidence in the different treatment groups.

Groups	No. of MPLs			Total no. of MPLs	No. of animals with MPLs/total no. of mice	Mean no. of tumors/MPLs	MPLs		
	Proximal	Middle	Distal	–			Incidence (%)	Burden	Multiplicity
CONTROL	NIL	NIL	NIL	NIL	NIL	–	NIL	NIL	NIL
VEHICLE	NIL	NIL	NIL	NIL	NIL	–	–	–	–
AOM/DSS	5	7	11	23	8/8	2.87 ± 0.599	100	2.87	2.87
AOM/DSS + SAHA	3	5	7	15	5/8	1.87 ± 1.536	62.5	1.87	3
AOM/DSS + SelSA-1	1	3	4	8	3/8	1 ± 1.322	37.5	1	2.66

MPL incidence = the percentage of animals having MPLs.

MPL burden = the total number of MPLs counted/ total number of mice.

MPL multiplicity = the total number of MPLs counted/ number of MPLs bearing mice.

The values are Mean ± S.D. of n = 7–8 animals from each group.

Further, histopathological examinations of colons of the AOM/DSS group showed clear carcinogenic changes such as disrupted epithelial barrier or epithelial erosion; surface ulceration with an increased influx of neutrophils into the lamina propria and sub-mucosa layer (shown by yellow arrows). Further structural distortion of the crypts, as well as widening of the gap between the crypt base, was evident in the case of AOM/DSS (shown by black arrows) compared to normal colonic histoarchitecture with crypts interspersed and connective tissues seen in the Control group animals^14,20^. Both SAHA and SelSA-1 significantly ameliorated these carcinogenic changes with the reduction in aberrant crypts and restoration towards normal colonic histoarchitecture shown in Fig. 3b(i–v), with SelSA-1 being more potent than SAHA demonstrating its exacerbated chemotherapeutical potential.

### Safety profiles of SelSA-1

**Figure 4:**
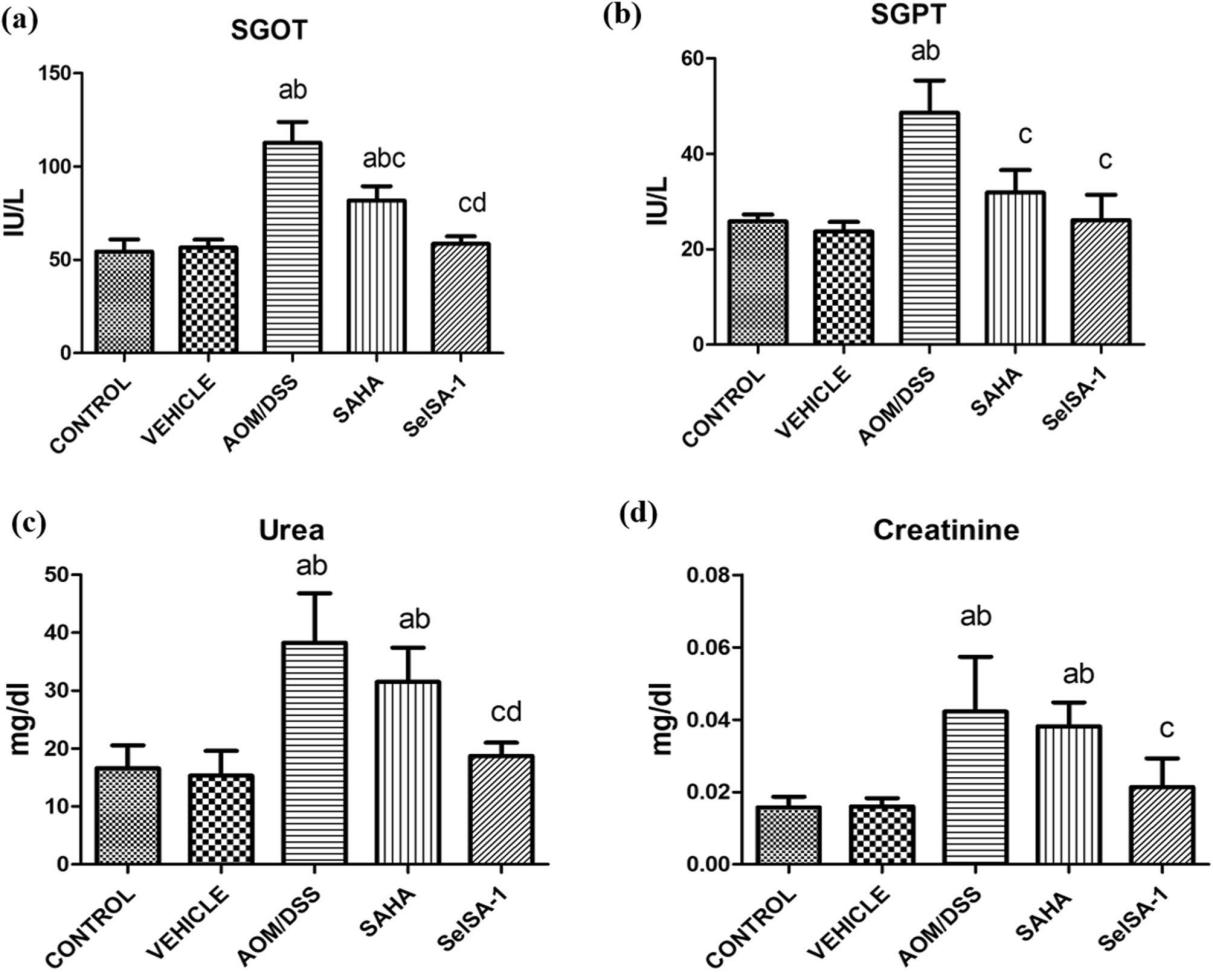
Graphical representation of hepatic and renal toxicity profiling of both SAHA and SelSA-1 on serum samples of the different treatment groups. (**a**) SGOT, (**b**) SGPT, (**c**) Urea, (**d**) Creatinine in different treatment groups. Data are expressed as mean **±** SD of at least 7–8 independent observations and analyzed using one-way ANOVA (Tukey multiple comparison method); where (**a**) represents p < 0.05 when compared between Control *vs* Vehicle, treated, AOM/DSS, AOM/DSS + SAHA, AOM/DSS + SelSA-1; (**b**) represents p < 0.05 when compared between Vehicle *vs* AOM/DSS, AOM/DSS + SAHA, AOM/DSS + SelSA-1; (**c**) represents p < 0.05 when compared between AOM/DSS *vs* AOM/DSS + SAHA, AOM/DSS + SelSA-1; (**d**) represents p < 0.05 when compared between AOM/DSS + SAHA *vs* AOM/DSS + SelSA-1.

Although SelSA-1 demonstrated enhanced chemotherapeutic potential in AOM/DSS-induced CAC, it was critical to evaluate the safety profiles at the concentrations used. Therefore, systemic toxicity was assessed using hepatic and renal function markers. Increased SGOT and SGPT along with increased urea and creatinine levels in AOM/DSS group were observed compared to Control and Vehicle-treated groups Fig. 4a–d. Contrary to this, decreased liver markers levels (p < 0.05), were established in the sheath of mice administrated with SAHA and SelSA-1 respectively compared to Group 3 i.e., AOM/DSS, indicating the sign of resolution from the classical sign of CAC without triggering any side effects on the normal physiology of the body. Similar to this, the levels of renal function markers were also found to be elevated in the case of the AOM/DSS group as compared to Control and Vehicle-treated group indicating renal toxicity in the animals administrated with AOM/DSS. On the other hand, there is a sign of normalization or resolution in the case of SAHA & SelSA-1 groups as shown in Fig. 4c and d. These findings indicated not only the enhanced anti-tumorigenic effects of SelSA-1 at lower dosages than SAHA but also better safety margins.

### Chemotherapeutic effects of SelSA-1 are mediated via ablation of oxidative stress

**Figure 5:**
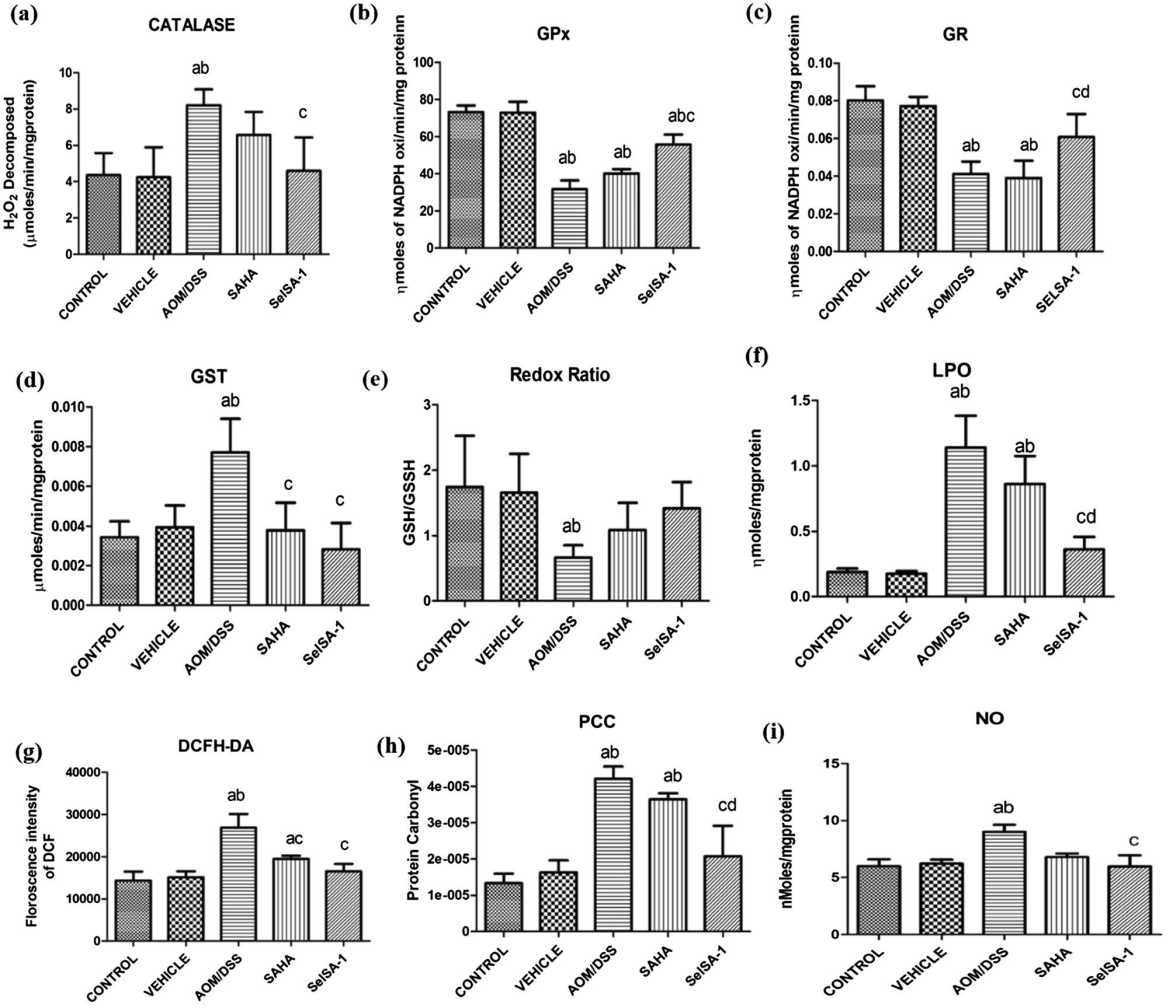
Graphical illustrations of enzymatic and non-enzymatic activities of (**a**) Catalase, (**b**) GPx, (**c**) GR, (**d**) GST, (**e**) Redox Ratio, (**f**) LPO, (**g**) DCFH-DA, (**h**) Protein Carbonyl Content (PCC), (**i**) Nitric Oxide Synthase (NO) in different groups. Data are expressed as mean _**±**_ SD of at least 7–8 independent observations and analyzed using one-way ANOVA (Tukey multiple comparison method); where (**a**) represents p < 0.05, when compared between Control *vs* Vehicle, treated, AOM/DSS, AOM/DSS + SAHA, AOM/DSS + SelSA-1; (**b**) represents p < 0.05 when compared between Vehicle *vs* AOM/DSS, AOM/DSS + SAHA, AOM/DSS + SelSA-1; (**c**) represents p < 0.05 when compared between AOM/DSS *vs* AOM/DSS + SAHA, AOM/DSS + SelSA-1; (**d**) represents p < 0.05 when compared between AOM/DSS + SAHA *vs* AOM/DSS + SelSA-1.

Plethora of studies have indicated the critical role of redox modulation in the pathophysiology of carcinogenesis and inflammation-associated insults. Moreover, it has been postulated that the anti-cancer and pro-resolution effects of Se are mediated through the redox modulation of vital inflammatory pathways. Therefore, SelSA-1 mediated alteration in both enzymatic and non-enzymatic markers of oxidative stress was evaluated as a plausible key event behind the anti-cancer mechanism of SelSA-1 Fig. 5a–i, depicts AOM/DSS group induced enhanced cellular oxidative stress in terms of decreased activities of antioxidant activities GPx, GR along with a concomitant increase (p ≤ 0.05) in levels of total GST, total ROS, LPO, protein carbonyls and NO when compared to the Control and Vehicle group (Fig. 5). Contrary to this, mice supplemented with SelSA-1 showed favorable modulation of oxidative markers with lowered levels of total ROS, lipid peroxides, protein carbonyls, and nitric oxide. Also, the activities of antioxidant enzymes were found to be comparable to normal Control suggesting sustained redox homeostasis and signifying redox modulatory activities of SelSA-1.

### SelSA-1 promotes cancer cell apoptosis

**Figure 6:**
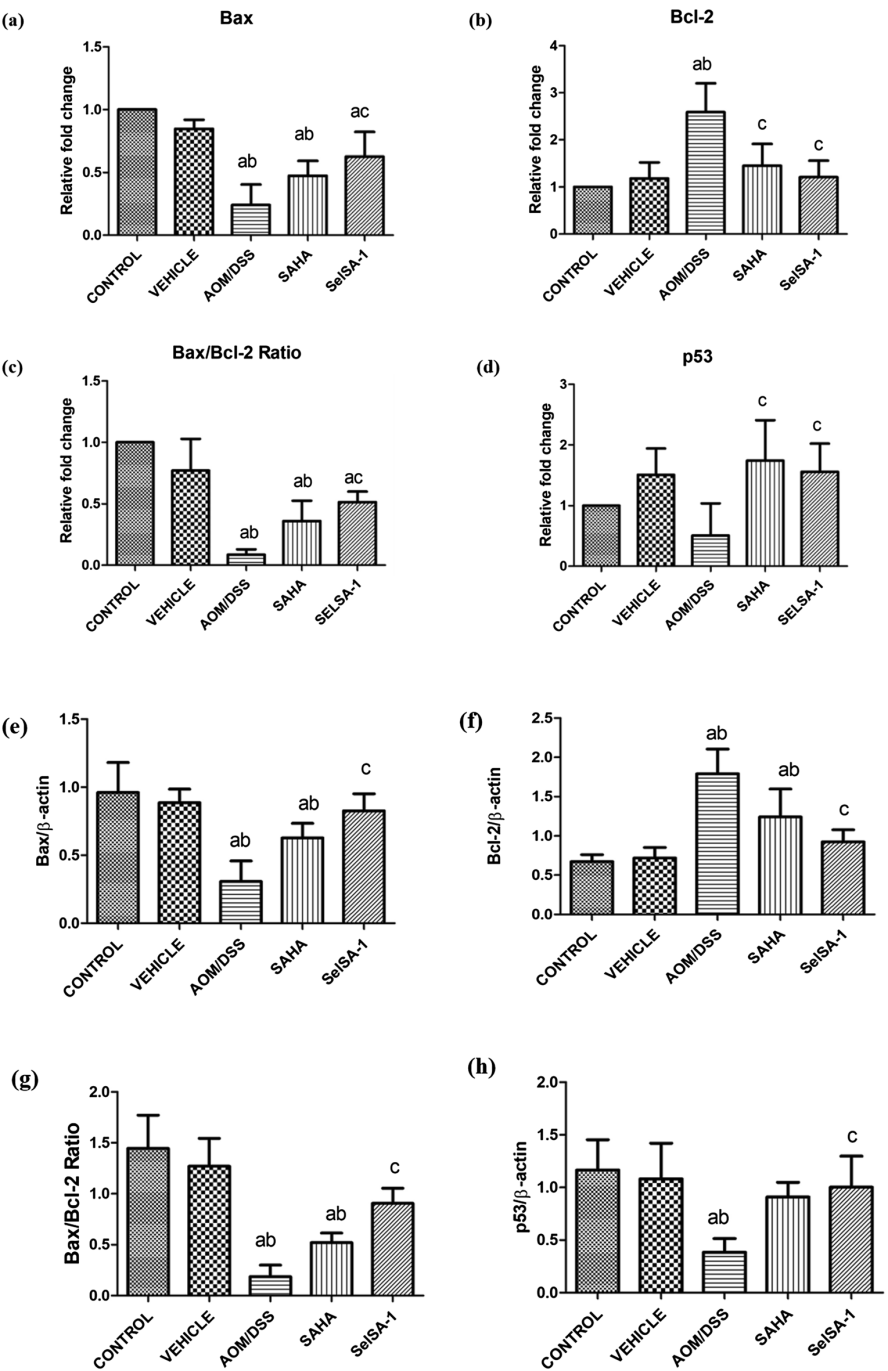
Alterations in the gene and protein expression profiling of different apoptotic factors (**a**, **e**) Bax (**b**, **f**) Bcl-2 (**c**, **g**) Bax/Bcl-2 Ratio (**d**, **h**) p53 in different treatment groups. Data are expressed as mean + SD of at least 4 independent observations and analyzed using one-way ANOVA (Tukey multiple comparison method); where (**a**) represents p < 0.05 when compared between Control *vs* Vehicle treated, AOM/DSS, AOM/DSS + SAHA, AOM/DSS + SelSA-1; (**b**) represents p < 0.05 when compared between Vehicle *vs* AOM/DSS, AOM/DSS + SAHA, AOM/DSS + SelSA-1; (**c**) represents p < 0.05 when compared between AOM/DSS *vs* AOM/DSS + SAHA, AOM/DSS + SelSA-1; (**d**) represents p < 0.05 when compared between AOM/DSS + SAHA *vs* AOM/DSS + SelSA-1.

Consistent with the above results a considerable alteration in a variety of crucial pro and anti-apoptotic factors such as p53, Bax, and Bcl-2 were compared through qPCR and indirect ELISA. Both SAHA and SelSA-1 showed remarkable (p ≤ 0.05) increases in p53, BAX, and p53 expression indicating enhanced apoptosis Fig. 6a–h. One of the most critical processes during carcinogenesis is the deregulation of the mitochondrial apoptosis pathway^21^. Bax and Bcl-2 are the principal members of the Bcl-2 family that play a vital role in tumor progression or suppression of intrinsic apoptotic pathways triggered by mitochondrial dysfunction^21^. As a result, the balance between pro- and anti-apoptotic factors can influence cellular fate. Contrary to this, the anti-apoptotic gene Bcl-2 was found to be decreased (p ≤ 0.05) in AOM/DSS-induced CAC group animals compared to SAHA and SelSA-1 validating this hypothesis. Also, we proposed the constructive effects of SelSA-1 compared to SAHA via Se incorporation as emerging evidence from epidemiological studies and clinical trials showing the beneficial anti-apoptotic and chemo-preventive effects of Se and Se-containing compounds^17,19^.

## Discussion

The role of a plethora of genetic, epigenetic, and cytogenetic pathways has been documented during carcinogenesis. Epigenetic regulations have emerged as key regulatory mechanisms in response to inflammation, trauma, and the transformation of normal cells to cancer cells^22–24^. These studies led to the exploration of HDAC inhibitors (HDACi) as potential therapeutic agents. Over the last few years, diverse HDACi (hydroxamic acid derivatives and non-hydroxamic HDACi) have been developed^25^. The first US-FDA-approved HDACi, SAHA (hydroxamic acid) showed promising chemotherapeutic effects against human carcinomas, myelomas, lymphomas, and neuroblastomas. However, due to its narrow safety margins and chemotherapeutic activities at the relevant doses limits its use for patients with cutaneous T-cell lymphoma (CTCL)^26^. Similarly, reduced potency, lack of stability, and unfavorable pharmacokinetic profiles limit the clinical use of other HDACi as well^26,27^.

Considering the ability of essential trace elements and a potent antioxidant Se to regulate HDACs in inflammatory, and cancer regulatory pathways^15,28^ a Seleno analog of SAHA (SelSA-1) was designed and evaluated for its chemotherapeutic effects in an experimental model of CAC. Similar derivatized analogs have been reported to regulate epigenetic-driven gene expression through the inhibition of HDACs in colon cancer^14,29^. Consistent with the earlier reports, SelSA-1 not only substantially reduced the neoplastic lesions in colons than its native compound SAHA^14^, but also ameliorated the colonic inflammation as seen in various classical hallmarks of colitis even at lower doses without prompting any side effects on the normal physiology of the body^29,30^. The favorable pharmacokinetic and safety profiles of SelSA-1 corroborate these findings^14,31^. Contrary to this the poor pharmacokinetics and severe toxicity of hydroxamic acid derivative HDACi has been reported owing to their ability to chelate zinc ions in the active site of HDAC through its CO and OH groups^18,32^. Additionally, lower IC_50_ of SelSA-1 than SAHA might be responsible for its enhanced chemotherapeutic efficacy in both primary colonocytes as well as NIH3T3 and HCT-115 colon cancer cell lines. Previously, lower IC_50_ of SelSA-1 than SAHA in lung cancer cell lines have been linked with enhanced cytotoxicity and efficacy^18^.

Since inflammation is a key component in tumorigenesis, the aggravated anti-inflammatory and chemotherapeutic potential of SelSA-1 might be attributed to the presence of Se in SelSA-1. An inverse association between physiological Se concentration and inflammation has been documented^33,34^. On similar lines, despite the known anti-inflammatory potential of SAHA^35–38^, the molecular mechanisms of SAHA and SelSA-1 remain unknown. Since both inflammation and tumorigenesis can have a redox basis^39,40^, the plausible involvement of SelSA-1-mediated redox modulation was studied. A large piece of scientific evidence suggesting oxidative insults as a key molecular event in CAC^41^ reiterates the possibility of our redox hypothesis as a possible mechanism for SelSA-1. Moreover, reported redox regulation of genetic and epigenetic mechanisms by Se^42–44^ further strengthens the possibility of redox modulation as a key mechanism behind the effective anti-carcinogenic effects of SelSA-1.

Previous reports from our laboratory hypothesized a multipronged mechanism of action of SelSA-1. One such mechanism involved the modulation of redox tone^14^, the induction of ER stress via p53 dependent mechanism comparable to trichostatin^43,45^, and another one through the differential binding on the catalytic sites of HDACs. The outcome of the present study is suggestive of SelSA-1-mediated amelioration of oxidative stress as one of the key anti-inflammatory and anti-tumorigenic mechanisms of SelSA-1. This redox modulatory activity of SelSA-1 might be due to (a) Se-mediated effects during the cellular metabolism of SelSA-1 or (b) the possibility of free SeH (thiols) being formed due to the reduction of Se dimer in SelSA-1^18^.

The Se-mediated effects are executed through the maintenance of physiological selenoprotein pools^5,15^. Currently, increased activity and expression of GPx, a well-known antioxidant selenoprotein and also a surrogate marker of Se levels in the body is suggestive of its role in mitigating oxidative stress^18^. Thus, considering the dynamic in vivo ROS scavenging potential of SelSA-1 discloses the stronger antioxidant and anti-inflammatory capacity due to the presence of Se in it, thus maintaining the redox homeostasis. Additionally, the free SeH active species formed through the reduction of Se dimer in SelSA-1 might also bind to the acetate groups and causes potent HDAC inhibitory activities^18^. A similar reduction of a disulfide bond in the cellular environment leading to the release of free thiol analog as the active species of HDACi known as FK228 has been reported^46^.

From an anti-cancer viewpoint, the balance between pro- and anti-apoptotic markers can determine cellular fate^21,47^. In this regard, one of the possible mechanisms is the modulation of p53-mediated signaling. Currently, SelSA-1 showed enhanced chemotherapeutic potential through the activation of p53-mediated apoptotic pathways involving pro-apoptotic (Bax) and anti-apoptotic (Bcl-2) genes. Since one of the most critical processes during carcinogenesis is the dysregulation of the mitochondrial apoptotic pathway^21^, SelSA-1 mediated modulation of these factors is indicative of regulation of cancer growth through activation of the apoptotic pathway promoted by mitochondrial dysfunction^48,49^. Notwithstanding the emerging epidemiological and clinical data indicating the similar constructive effects of Se and Se-containing compounds^17,19^, these apoptotic effects of SelSA-1 might work through a redox modulatory as reported currently or through a p53 dependent mechanism equivalent to trichostatin^43,45^. However, detailed mechanistic studies are commenced to ascertain the establishment of diverse chemotherapeutic pathways involved with its better anticancer potential and pro-resolving effects.

## Conclusion

In conclusion, the current study demonstrates a clear synergistic and enhanced redox modulatory/anti-inflammatory and chemotherapeutic effects of SelSA-1 compared to 2nd generation HDACi, SAHA. Further, mechanistic studies are currently underway to explore the translational feasibility of SelSA-1 as an effective chemotherapeutic in the future.

## Methods

### Chemicals

All the chemicals used in the present study were of analytical grade (AR) and were purchased from Sigma (India), HiMedia (India), SRL (India), and MP Biomedical (India). Azoxymethane (AOM) and Dextran Sodium Sulfate (DSS) used for CAC induction were procured from Sigma (India) and, MP Bio. (India) respectively. Similarly, Vorinostat used as a commercially available HDACi drug was purchased from TCI (India). Oxidized glutathione (GSSG), bovine serum albumin (BSA), reduced glutathione (GSH), glutathione reductase (GR), thio barbituric acid (TBA), sodium acetate, and NADPH used in the study were obtained from Hi-Media Laboratories Private Limited (Mumbai, India). Toxicity profiling was done using various Reckon Diagnostic Kits (Gujarat, India). Primary and Secondary Antibodies for protein expression profiling were purchased from Santa-Cruz (India).

### In vitro studies

#### Cell lines and culture conditions

Human fibroblast cell line (NIH-3T3), and Human colorectal cell line (HCT-115) were from National Centre for Cell Science (NCCS) Pune, Maharashtra, India, maintained in RPM I media supplemented with 10% FBS and humidified at 37 °C and 5% CO_2_. 1 × 10^4^ cells were seeded in each well of a 96 well plate containing both the drugs i.e., SAHA and SelSA-1 at different concentration (0.25 µM, 1.25 µM, 2.5 µM, 5 µM, 7.5 µM, 10 µM, 12.5 µM, 15 µM, 17.5 µM). The medium was aspirated after incubation for 24 h at 37 °C.

Similarly, colonocytes were obtained from the freshly isolated colonic segments of mice administered with AOM/DSS, by the method of Mouille et al.^50^ using the everted sacs method. Afterward, the isolated colonocytes were resuspended in RPM I media supplemented with 10% FBS and humidified at 37 °C and 5% CO_2_ for cytotoxicity assay.

#### Cytotoxicity assay (MTT)

The cell metabolic activity was measured using MTT (3-[4,5-dimethylthiazol-2-yl]-2,5 diphenyl tetrazolium bromide) assay as described earlier^50,51^. Briefly, NIH3T3 and HCT-115 cells were seeded with a cell count of 1 × 10^4^ cells per well, along with the different concentrationsof SAHA and SelSA-1 (0.25 µM, 1.25 µM, 2.5 µM, 5 µM, 7.5 µM, 10 µM, 12.5 µM, 15 µM, 17.5 µM). After 24 h, 20 µl MTT (5 mg/mL) was added to each well followed by incubation for 5 h, and the formazon crystal so formed were dissolved using DMSO. The absorbance was read at 570 nm using multimode plate reader BioTek Synergy H1, (United States), and percentage cell viability, as well as IC_50_ values of SelSA-1 and SAHA, were calculated. Control and Blank were also run simultaneously.

### In vivo studies

#### Animal procurement

Balb/c mice in the body weight range of 25–30 g were procured from the Central Animal House, Panjab University (Chandigarh, India). All animals were housed in the departmental animal room in polypropylene cages with 12 h light and dark cycles in temperature-maintained rooms. These animals received food (rodent chow) and water ad libitum. Animals were acclimatized for 1 week before the start of treatment and then assorted randomly in different groups and studies were carried out following the guidelines of The Committee for the Purpose of Control and Supervision of Experiments on Animals (CPCSEA), Government of India. Also, all the methods reported are in accordance with ARRIVE guidelines. The study was approved by the Institutional Animal Ethical Committee (IAEC) of Panjab University, with Approval No. PU/49/99/CPCSEA/IAEC/2019/270.

### Experimental model and design

A chemically (Azoxymethane (AOM)/Dextran sulfate sodium (DSS)) induced experimental model of CAC was used to study the chemotherapeutic effects of SelSA-1. The Balb/c mice were randomly divided into various groups as described below, ensuring that each group'’ average body weight was the same at the start of the experiments.

#### Group 1: Control group

The animals were kept on a normal diet and water ad libitum.

#### Group 2: Vehicle treated

Animals were administered with normal drinking water for 11 weeks, followed by 2% DMSO (vehicle for drugs) intraperitoneally (IP) daily for 21 days.

#### Group 3: AOM/DSS group

**Figure 7:**
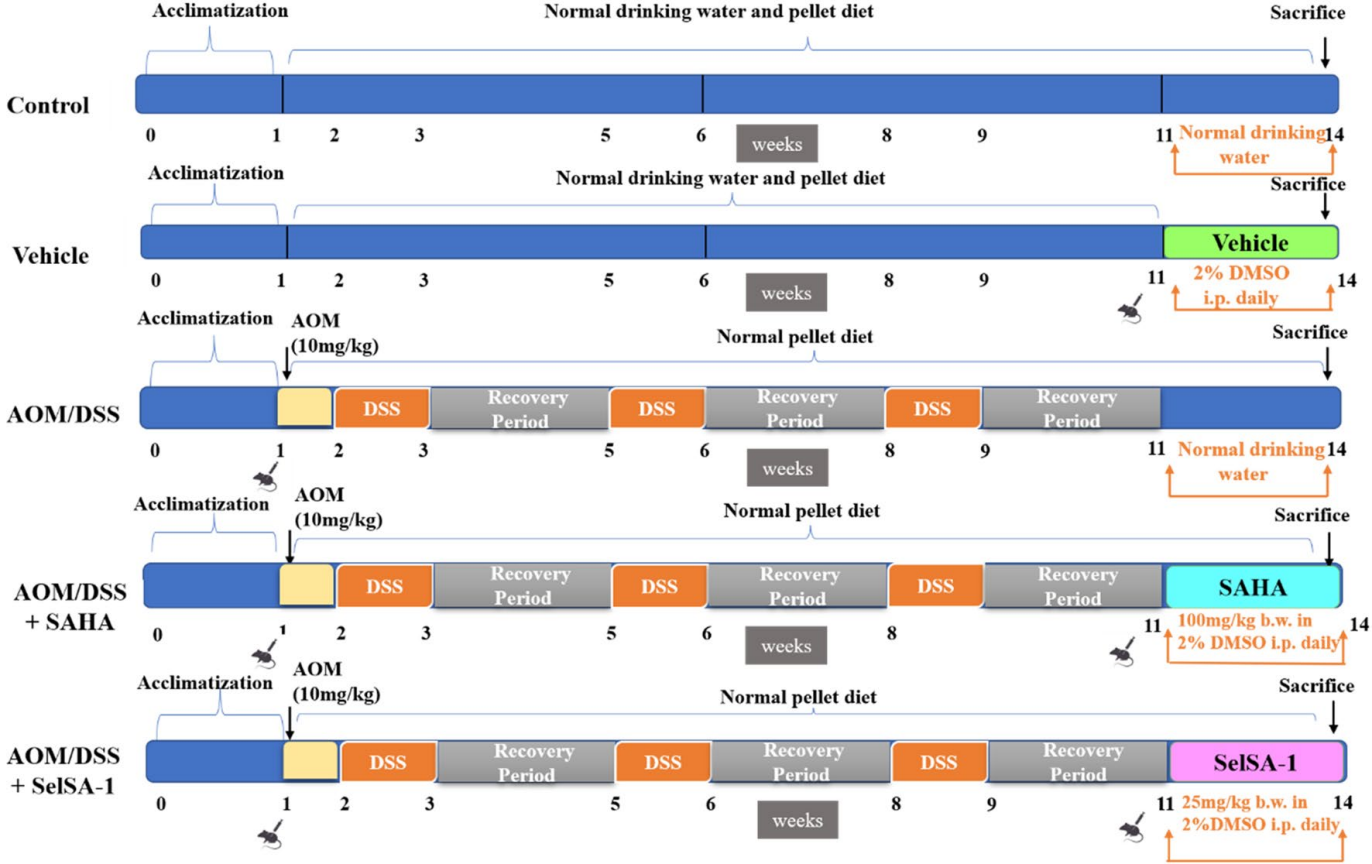
Schematic timeline representation for AOM/DSS-induced colitis-associated colorectal cancer model (CAC).

CAC model was established using AOM/DSS as per protocol^52^ discussed below. On day 0, the baseline weight of the mice was recorded, and each mouse was given a single AOM dose (10 mg/kg) once during the cycle intraperitoneal (IP), freshly prepared in 1 × Phosphate Buffer Saline (PBS) buffer (pH-7.2). After 1st week of the AOM administration, three (3) cycles of DSS were given as shown below in Fig. 7. During each DSS cycle, mice received 2.5% (2.5 g/100 mL) DSS solution for 7 days in drinking water (distilled) followed by standard drinking water for about two weeks also called a recovery period. The DSS solution was replaced in clean bottles three times (every 2–3 days) during each cycle. Regular weighing was done as mice can lose significant body weight after DSS administration. After the successful completion of 11 weeks, a couple of animals from both groups were randomly selected. The colonic sections were isolated and histological studies were carried out to ensure the successful establishment of tumors in AOM/DSS group animals compared to the Control group (Fig. 7).

After confirmation of the CAC model establishment, 2/3rd animals from AOM/DSS group were randomly selected and equally divided for treatment with SAHA or SelSA-1, respectively, as described below. In contrast, the remaining 1/3rd number of animals were provided with 2% DMSO (vehicle for SAHA and SelSA-1) for 21 days before dissection.

#### Group 4: AOM/DSS + SAHA

After 11 weeks of AOM/DSS treatment, animals were administered with SAHA for 21 days at a dose of 100 mg/kg body weight (IP), dissolved in 2% DMSO^12,14^.

#### Group 5: AOM/DSS + SelSA-1

After 11 weeks of AOM/DSS treatment, animals were administrated with SelSA-1 for 21 days at a dose of 25 mg/kg body weight (IP), dissolved in 2% DMSO^14^.

### Toxicity analysis by liver and renal function test

Blood was drawn from the retro-orbital plexus of mice with a fine sterilized glass capillary and was allowed to clot when left undisturbed for about 2 to 3 h. at 37 °C. Thereafter, serum was prepared from the clotted blood by centrifugation at 1500 rpm for 10–15 min at 4 °C. Various serum biochemical assays for liver function assessment such as Serum glutamic pyruvic transaminase (SGPT), Serum glutamic oxaloacetic transaminase (SGOT), Creatinine, and Urea levels were measured using REKON ENZOPAK diagnostic kit as per the manufacturer's protocol.

### Anti-inflammatory studies

Myeloperoxidase activity as a biochemical measure of inflammation was measured in the colonic tissue according to the method^53^.

### Morphological indicators of colitis

Changes in the body weights, mortality rates, colon lengths, and weight and disease activity index (DAI) were used as surrogate morphological markers to establish the onset of colitis. The baseline weight of each mouse from the respective groups was logged and the mortality rate was recorded on the animal that loses 25% of the total initial weight. Based on these observations, the survival records were maintained at regular intervals, and these animals were not included in further studies. Also, gross signs of colitis other than weight loss, such as colon weight, and length were monitored in animals present in different groups. Changes in the colon length was the classical hallmark of cancer. So, any change in the colon will be the marker for the diseased condition.

### Evaluation of chemotherapeutic effects of SAHA and SelSA-1

The colons were removed and flushed down with ice-cold physiological saline (NaCl solution, 9 g/L) and then opened longitudinally along the median and laid flat to observe for the incidence of macroscopic neoplastic lesions/plaques called the multiple plaque lesions (MPLs) or presence of any tumors. The colons were divided into proximal, medial, and distal segments for the morphological examination^54^. The morphological parameters such as tumor incidences, tumor burden, and tumor multiplicity were used to evaluate and compare the chemotherapeutic index of SAHA and SelSA-1^14^.

### Histological examination

5–7 µm thick wax-embedded sections of formalin-fixed colonic segments were prepared using a hand-driven microtome. Permanent mounts were prepared and stained with hematoxylin and eosin using a standard protocol and viewed under the light microscope. The slides were analyzed, scored, and photomicrographs from different treatment groups were clicked at 10 × magnification.

### Estimation of redox modulation

The redox modulatory effect was measured using Enzymatic and Non-Enzymatic markers of oxidative stress in 10% of colonic homogenates prepared in RIPA buffer (pH 7.4).

#### Non-enzymatic markers of oxidative stress

Determination of total ROS levels in the colon tissue was based on the modified method of Driver et al.^55^, using the fluorescent probe DCFH-DA.

Similarly, the extent of lipid peroxidation (LPO), Protein Carbonyl Content (PCC), Nitric Oxide Synthase (NO), and Redox Ratio (GSH/GSSG) were measured in freshly prepared colonic homogenate by following the respective methods^56–59^.

#### Enzymatic markers of oxidative stress/antioxidant enzymatic activities

The specific activities of antioxidant defense enzymes such as Catalase, Glutathione peroxidase (GPx), Glutathione reductase (GR), and Glutathione-S-Transferase (GST) were estimated by the methods described^60–63^ respectively after normalization with total colonic protein levels measured via Lowry method^64^.

### Gene and protein expression studies

The chemotherapeutic efficacy of SAHA and SelSA-1 were compared by studying the gene and protein expression profiles of apoptotic factors viz p53, Bax, and Bcl-2.

#### Quantitative real-time PCR (qPCR)

Total RNA was isolated from colon tissues using TRI-reagent as per the manufacturer’s protocol. After evaluating the concentration and purity of RNA, cDNAs were synthesized and subjected to qPCR- based amplifications of apoptotic genes like p53, Bax, and Bcl-2 on a Step One Real-Time PCR System (Applied Biosystems), using a specific primer set as follows Bax primer pair: Forward 5′-AGGATGCGTCCACCAAGAAGCT-3′ and Reverse sequence 5′-TCCGTGTCCACGTCAGCAATCA-3′; Bcl-2 primer pair: Forward 5′-CCTGTGGATGACTGAGTACCTG-3′ and Reverse Sequence 5′-AGCCAGGAGAAATCAAACAGAGG-3′; p53 primer pair: Forward 5′-ACCGCCGACCTATCCTTACC-3′ and Reverse Sequence 5′-TCTTCTGTACGGCGGTCTCTC-3′ and, β-actin primer pair: Forward 5′-GGGACCTGACGGACTAC-3′ and Reverse Sequence 5′-TGCCACAGGATTCCATAC-3′. Relative quantification of target gene expression was normalized concerning the average Ct value of the housekeeping gene i.e., β-actin. All data were expressed as mean ± SD of at least n = 4 independent observations^15^.

The protein expressions of pro/anti-apoptotic mediators such as p53, Bax, and Bcl-2 were studied via indirect ELISA, using specific primary and peroxidase-labeled secondary antibodies as described in the method of^19^. The protein expression was assessed using an ABTS and H_2_O_2_-based spectrometric detection system where the normalization was achieved using β-actin protein as an internal control.

### Statistical data analysis

All data were expressed as mean ± SD of at least n = 6–8 independent observations. An unpaired, two-tailed “t” test is used to compare the mean for various treatment groups with Control, and the statistical significance of the data was determined using one-way ANOVA (Turkey multiple comparison method) to compare various treatment groups using GraphPad Prism 5.0 program (GraphPad Software, San Diego, CA)., and RStudio 2022.07.0 Build 548. A p-value ≤ 0.05 was considered statistically significant.

## Supplementary Information

Supplementary Figure S1.

## Data Availability

The authors declare that all data supporting the study's findings are included in the article.
