# Supplementary material for: SelSA-1, a novel HDAC inhibitor demonstrates enhanced chemotherapeutic potential by redox modulation
Source: Sci Rep. 2023 Jun 8;13:9301. doi: 10.1038/s41598-023-36555-w (PMC10250299; doi:10.1038/s41598-023-36555-w)
Supplement: Supplementary file 1 — Supplementary Figure S1. [file 41598_2023_36555_MOESM1_ESM.docx]

**Supplementary File**

**S1)** Curve fit plot of percent cell viability against various concentrations of SAHA and SelSA-1 for determination of IC_50_ value

**
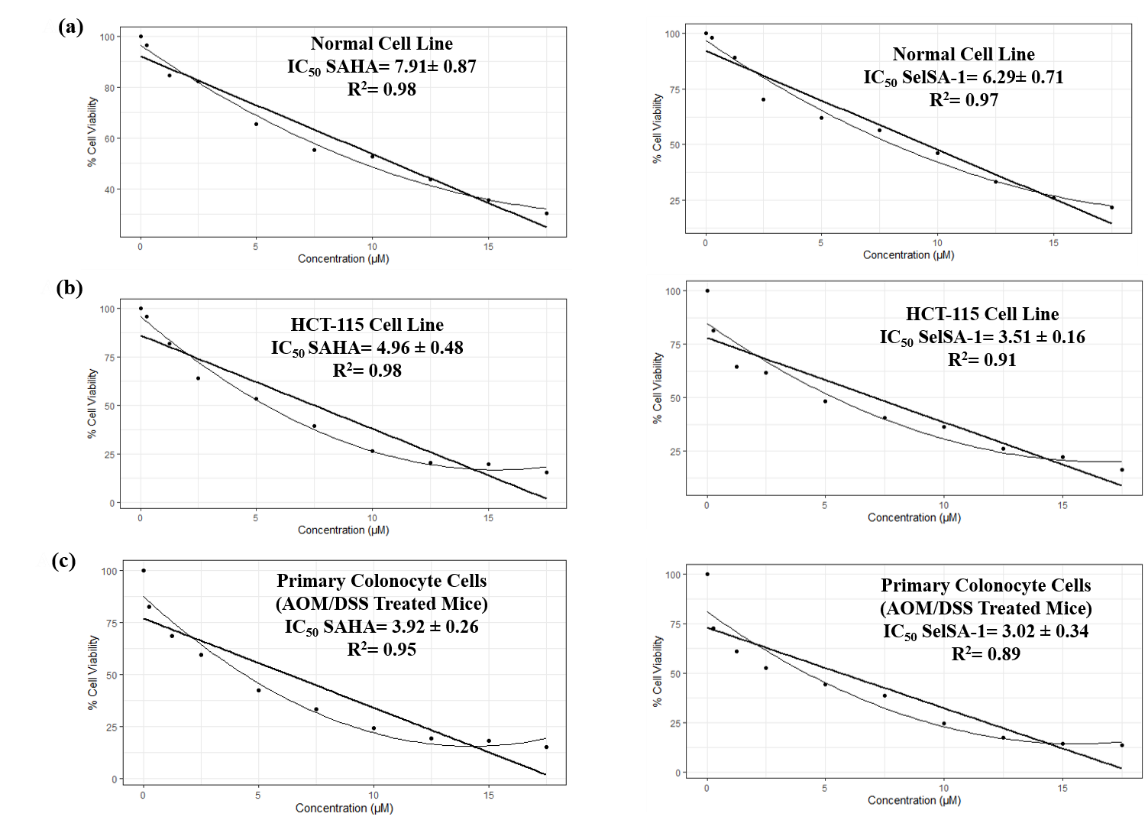
**

**Supplementary Figure S1)**: **IC_50_ values of SAHA and SelSA-1 against** **(a)** NIH3T3 (Normal cell line) **(b)** HCT-115 (Colon cancer cell line) and **(c)** Primary Colonocytes cells are the primary cells isolated from AOM/DSS treated mice using curve fit plot. A μM range of SAHA and SelSA-1 drug concentrations was examined for 24h to determine respective IC_50_ values. RStudio 2022.07.0 Build 548 was used to plot these graphs.
